# Ingestion, Immunity, and Infection: Nutrition and Viral Respiratory Tract Infections

**DOI:** 10.3389/fimmu.2022.841532

**Published:** 2022-02-28

**Authors:** Coen Govers, Philip C. Calder, Huub F. J. Savelkoul, Ruud Albers, R. J. Joost van Neerven

**Affiliations:** ^1^ Cell Biology and Immunology, Wageningen University and Research, Wageningen, Netherlands; ^2^ School of Human Development and Health, Faculty of Medicine, University of Southampton, Southampton, United Kingdom; ^3^ National Institute for Health Research (NIHR) Southampton Biomedical Research Centre, University Hospital Southampton National Health Service (NHS) Foundation Trust and University of Southampton, Southampton, United Kingdom; ^4^ Nutrileads BV, Wageningen, Netherlands; ^5^ Research & Development, FrieslandCampina, Amersfoort, Netherlands

**Keywords:** infection, immunity, nutrition, infant, elderly, respiratory virus

## Abstract

Respiratory infections place a heavy burden on the health care system, particularly in the winter months. Individuals with a vulnerable immune system, such as very young children and the elderly, and those with an immune deficiency, are at increased risk of contracting a respiratory infection. Most respiratory infections are relatively mild and affect the upper respiratory tract only, but other infections can be more serious. These can lead to pneumonia and be life-threatening in vulnerable groups. Rather than focus entirely on treating the symptoms of infectious disease, optimizing immune responsiveness to the pathogens causing these infections may help steer towards a more favorable outcome. Nutrition may have a role in such prevention through different immune supporting mechanisms. Nutrition contributes to the normal functioning of the immune system, with various nutrients acting as energy sources and building blocks during the immune response. Many micronutrients (vitamins and minerals) act as regulators of molecular responses of immune cells to infection. It is well described that chronic undernutrition as well as specific micronutrient deficiencies impair many aspects of the immune response and make individuals more susceptible to infectious diseases, especially in the respiratory and gastrointestinal tracts. In addition, other dietary components such as proteins, pre-, pro- and synbiotics, and also animal- and plant-derived bioactive components can further support the immune system. Both the innate and adaptive defense systems contribute to active antiviral respiratory tract immunity. The initial response to viral airway infections is through recognition by the innate immune system of viral components leading to activation of adaptive immune cells in the form of cytotoxic T cells, the production of neutralizing antibodies and the induction of memory T and B cell responses. The aim of this review is to describe the effects of a range different dietary components on anti-infective innate as well as adaptive immune responses and to propose mechanisms by which they may interact with the immune system in the respiratory tract.

## Introduction

Due to the increasing population density and the worldwide trend of urbanization, people are living closer together, thus providing a favorable environment for transmission of respiratory viruses and other infective organisms. Contacts with animals such as chickens and pigs, but also with wild animals, increase the risk of pandemic zoonotic disease outbreaks. Examples include the 2009 swine influenza pandemic and the current severe acute respiratory syndrome coronavirus 2 (SARS-CoV-2) pandemic. COVID-19, caused by SARS-CoV-2, is a reminder of the impact that respiratory viral infections can have on human health and wider society.

Respiratory infections are common in the elderly and in very young children and place a heavy burden on the health care system, especially in the winter months. In addition to the direct impact of respiratory infections, there are causal links between respiratory infections and the development and worsening of chronic respiratory diseases such as asthma in children (1–3), long COVID in children and adults (4–6), COVID-19 induced pulmonary fibrosis in adults and the elderly (7), and chronic obstructive pulmonary disease (COPD) in the elderly (8).

It is therefore highly relevant from a societal perspective, in addition to developing effective vaccines and new antiviral treatments, to understand more about possible preventive, lifestyle-related approaches, including those related to nutrition. Therefore, it is important to understand the possible role that nutrition can play in reducing the occurrence and in dampening the severity of these infections. This role has been largely neglected to date, even though there are indications that nutrition in general, and specific nutrients and other food components in particular, may improve resistance against viral infections. In this review we will describe which population subgroups are susceptible to respiratory infections, which immunological mechanisms contribute to antiviral immunity, how nutritional components can modulate antiviral immune responses and infections, and finally we will propose how these components that are ingested through the gastrointestinal tract can have effects on immunity in the respiratory tract.

## Immune Vulnerability: The Young and Old

**Figure 1:**
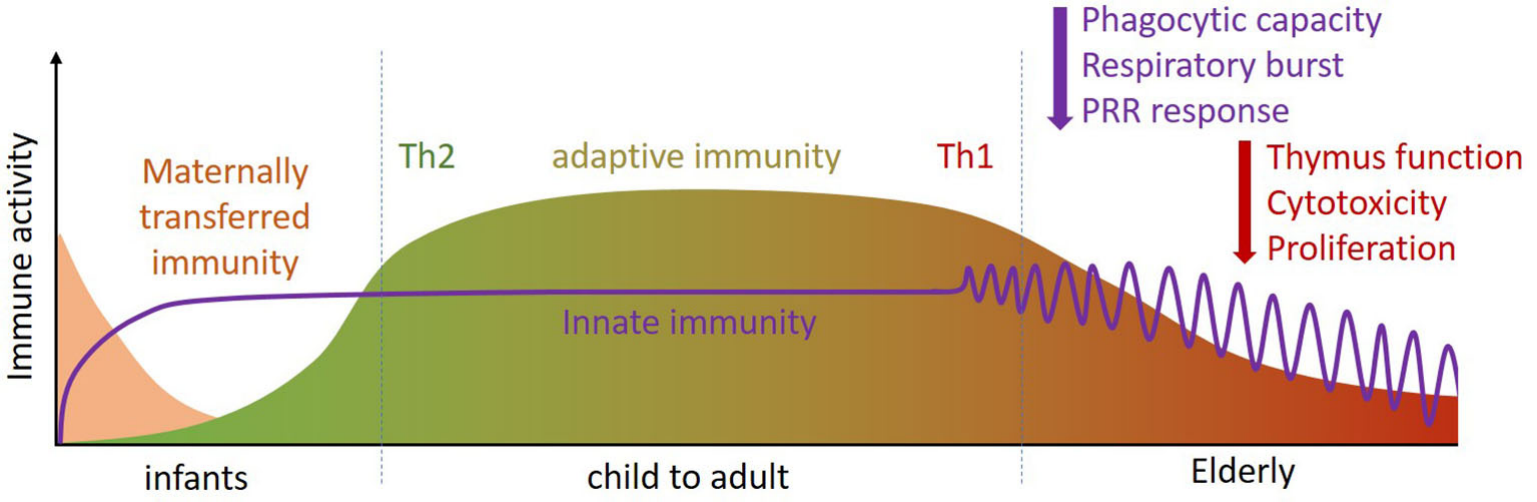
Throughout the life course (infant, child to adult, elderly) the immune system changes in composition and activity. Innate immunity is already present at birth and quickly matures (purple line). For adaptive immune defense infants rely on placental transfer and mothers’ milk for their first period (pink section). During development, the adaptive immune system phenotype changes from Th2 dominant [i.e. tolerant (green)] to Th1 dominant [i.e. inflammatory (red)]. At later age, specific functions of innate and adaptive immunity become less efficient (purple and red arrows). Th, T helper.

Both young children (0-4 years) and the elderly (> 65 years) are more susceptible to respiratory and gastrointestinal infections than older children and young and middle aged adults (9, 10) as their immune system is less effective (11). The underlying reasons for this differ between young children and the elderly. In young children, lower immune responsiveness results from a less matured adaptive immune system, especially in the first 2-3 years of life, which cannot always respond well to external stimuli. Moreover, the type of adaptive immune response in early life is more focused on the prevention of inflammation, as a result of which Th1 immunity and pro-inflammatory responses are not yet well developed [(11, 12) and **Figure 1**]. The innate immune system, however, is already developed in young children. Pan-pathogen receptors such as toll-like receptors (TLRs) and other pattern recognition receptors (PRRs) are present soon after birth, enabling recognition and responses by innate immune cells in the form of antiviral cytokine production after infection (11, 12). Protection of infants against infection is further provided by transplacental transport of immunoglobulins during pregnancy, and by breastfeeding after birth. In addition to immunoglobulins, breastmilk contains a wide range of immune-related components including micronutrients, oligosaccharides, antimicrobial proteins, and immunoregulatory cytokines that support immune maturation and regulation (13–15), as well as the development of the microbiota in early life [(16, 17) and **Figure 1**].

The reduced immune responsiveness/efficacy in the elderly, termed immune senescence, is characterized in particular by a less flexible adaptive immune response [(18–22) and **Figure 1**]. One contributor to immune senescence is likely to be the decreased output of immune cells from bone marrow, the site of origin of all immune cells, with increasing age. Furthermore, involution of the thymus with age decreases output of naive T cells, resulting in reduced capacity to respond to new antigens. In addition to altered numbers of immune cells in the circulation, their function is often impaired in older people. For example, neutrophils show impaired phagocytosis, respiratory burst and bacterial killing; natural killer (NK) cells have impaired cytotoxicity towards virally-infected and tumor cells; dendritic cells (DCs) have impaired responsiveness to immune signals. T cells have reduced ability to proliferate and to produce important cytokines like interleukin (IL)-2 and interferon (IFN)-γ; Cytotoxic T cell activity is reduced and antibody production by B cells is altered. Hence, older people can show a broad range of immune impairments, making them more susceptible to infectious diseases. This includes respiratory illnesses caused by viruses. Immune senescence also impairs responses to vaccination, including to the seasonal influenza vaccine (23, 24).

The reduced adaptive immune response to vaccination and to viral infection in the elderly may resemble the reduced adaptive immune response in young children, but it has a different cause. In infants and toddlers up to 2-3 years of age, T-cell responses and the induction of specific antibodies are reduced because of immaturity of the immune system; in elderly, there is a bias towards generation of memory T cells, and the ability to mount naive T cell responses to new antigens, as well as vaccines, is reduced.

Whereas even at a few weeks of age, infant already have sufficient TLR expression and responsiveness to TLR ligands like single stranded RNA (11) which induce IFN-α, the elderly have a much decreased IFN-α response to influenza viruses (25). This has been shown in particular for TLR7 and TLR8, the receptors that are important in protecting against single stranded RNA viruses, to which most respiratory viruses belong. For example, in older women, the plasmacytoid dendritic cell (pDC) response to TLR7 stimulation with R848 is notably decreased (26). In this context it is interesting to note that in COVID-19 patients with a severe clinical picture, two different TLR7 variants were found to be associated with reduced antiviral function (27).

The immune vulnerability in the very young and the elderly thus illustrates the need for supporting antiviral immune function, for example by nutritional intervention. But let’s first discuss the mechanisms of antiviral immunity.

## Mechanisms of Antiviral Immunity

The human immune system comprises an innate and adaptive component. The innate, or inborn, component is rapid (responds minutes to hours after infection), reacts to a large variety of pathogens and is important in the primary protection against infection, but is also limited in effectiveness. The adaptive immune system on the other hand is slow to respond (days to weeks), but highly specific and effective. In fact, the adaptive system is activated by its innate counterpart *via* the antigen presenting capacity of monocytes, macrophages and DCs.

Both the innate and adaptive defense systems contribute to active antiviral immunity. The initial response against viral airway infections is through sensing of viral replication in infected host cells by cells of the innate immune system *via* PRRs such as TLR7/8 and RIG-I. PRRs recognize repetitive evolutionarily conserved structures on potential pathogens, such as bacterial cell wall components (lipopolysaccharides, peptidoglycans) and sugars, and also intracellular components of pathogens such as DNA and RNA. The best studied PRRs are the TLRs, of which TLR7 and TLR8 are particularly important against single stranded RNA viruses, such as influenza virus, respiratory syncytial virus (RSV), rhinovirus and SARS-CoV-2. They recognize single stranded RNA that is not found in endosomes under normal metabolic activities of human cells. The recognition of pathogenic structures such as single stranded viral RNA by TLR7 and TLR8 results in induction of anti-viral type I and type III interferons that have a direct inhibitory effect on viral replication. In addition, these interferons (α, β and λ) cause nearby cells to heighten their anti-viral defenses, inducing an antiviral state. The innate defense system includes monocytes, myeloid and plasmacytoid DCs (mDCs and pDCs), macrophages, NK and natural killer T (NKT) cells, and neutrophils, eosinophils, and basophils. Especially the pDCs are high producers of type I interferons that play a key role in antiviral immunity (28–30) to shape the local immunological environment in order to eliminate viruses.

In addition to a first broad response and activation of the adaptive immune system, specific components of the innate immune system can also directly target respiratory pathogens. For example, NKT cells play a direct role in antiviral immunity (31–33). They have receptors that can recognize lipid when presented on major histocompatibility complex (MHC)-like molecules such as CD1c on innate immune cells. In addition, iNKT cells can inhibit the influenza A-induced immunosuppressive activity of myeloid suppressor cells (34) and iNKT activation can shorten influenza infection (35). The finding that milk contains lipids known as ligands for NKT cells (36) may indicate that the milk lipid fraction also plays a role in antiviral immunity, as well as in intestinal homeostasis as NKT cells also recognize lipids from the intestinal microbiota (37, 38). Plant lipids can also activate NKT cells (39).

Respiratory and gastrointestinal epithelial cells - which together with mucus form a first physical barrier against infection - can also contribute to antiviral immunity. Epithelial cells express TLRs and other PRRs such as RIG I and recognize viral components. In response, both intestinal epithelial and respiratory epithelial cells can produce antiviral IFNs (40–42).

The adaptive defense system consists of the B lymphocytes, which can produce antibodies against viruses, and both the CD4+ and CD8+ T lymphocytes, which can recognize protein fragments of the virus presented by MHC molecules on innate immune cells. This adaptive immune response is crucial in protecting against re-infection with the same pathogen through its long-lasting immunological memory. However, the adaptive immune response starts slowly and it takes a few days to weeks after infection before antibodies become detectable. This results from the process in which innate immune cells such as macrophages or DCs move from the site of infection to nearby specialized immunological compartments such as lymph nodes or the spleen. Monocytes, macrophages and DCs collect pathogenic peptide fragments and present those on MHC molecules - or human leukocyte antigens (HLA) in humans - to T cells that specifically recognize the MHC-peptide complex, resulting in T cell activation and instruction of antigen-specific B cells. After activation, B cells and T cells mature and proliferate (clonal expansion) before they can either start producing antibodies or migrate to the site of infection to kill infected host cells, respectively. Maintenance of virus-specific T and B cells provides a memory giving protection against reinfection. This is the basic principle underlying pathogen-specific protection by adaptive memory induced by vaccination.

Passive immunity against virus infections can be provided by antiviral antibodies already circulating in the blood that can neutralize the virus, but there are also small molecules such as complex sugars (oligosaccharides) and oligosaccharide-containing sphingolipids that can also exert such a neutralization role (43–46).

Another way the innate immune system confers protection against infections has been revealed through vaccination with BCG (tuberculosis vaccine). Surprisingly, BCG vaccination was found to also result in better protection against other pathogens (47, 48). Although this can be partly explained by cross-reacting antibodies which can bind to more than one pathogen, it has recently been shown that the innate immune system also has a certain degree of memory. This so-called “innate immune memory” or “trained immunity” appears to result from epigenetic modifications in the DNA conferring innate immune system cells with the ability to respond more strongly to stimulation by pathogens *via* TLRs and exhibiting some degree of cross-protection (49–52). It has recently been postulated that “trained immunity” may also protect against viral respiratory infections such as COVID-19 (53), and this concept is currently being investigated in multiple studies (54).

## Nutrition and the Immune System

Foods and beverages provide macro-and micronutrients, as well as other bioactive components, that contribute to the normal functioning of the immune system, including supporting barrier function. The impact of several food components such as specific vitamins and minerals, and also proteins, flavonoids, and non-digestible polysaccharides, as well as probiotics and short-chain fatty acids, on the immune system has recently been comprehensively reviewed (55, 56). These effects are relevant to anti-viral immunity (57).

Food components act in a variety of ways to influence immune responsiveness against acute upper respiratory tract infections by viruses:

- Macronutrients act as fuels for energy generation by immune cells;
- Macronutrients provide substrate (“building blocks”) for the biosynthesis that is involved in the immune response (e.g. amino acids for immunoglobulins, cytokines, new receptors, acute phase proteins);
- Many micronutrients are regulators of molecular and cellular aspects of the immune response (e.g. iron, zinc, vitamin A, vitamin D);
- Some nutrients are substrates for the synthesis of chemicals involved in the immune response (e.g. arginine and nitric oxide; arachidonic acid and eicosanoids);
- Some micronutrients have specific anti-infection roles (e.g. zinc, vitamin D);
- Some proteins play a direct role in pathogen clearance and immune function (e.g. breast milk proteins like IgG, lactoferrin, and lysozyme);
- Many nutrients and bioactives are involved in protection of the host from the oxidative and inflammatory stress imposed by the immune response (e.g. vitamin C, vitamin E, cysteine, zinc, copper, selenium, flavonoids);
- Many food components contribute to creating a diverse microbiota that supports the immune response (e.g. plant-derived fibers and non-digestible polysaccharides, prebiotic oligosaccharides and human milk oligosaccharides);
- Probiotic bacteria that produce metabolites like short chain fatty acids (SCFAs) and some vitamins, support barrier function and contribute to immune support;
- Some food components act as aryl hydrocarbon receptor ligands, acting to strengthen barrier function (e.g. quercetin).

These considerations suggest multiple sites of interaction of food components with the immune system. Firstly, absorbed food components can act systemically to target the different components of the immune system (e.g. in bone marrow, the thymus, the bloodstream, secondary lymphoid organs, and other organs). Secondly, multiple food components can act to influence the immune system without being absorbed systemically. For example, they could have local actions on epithelial barrier function or on the gut-associated lymphoid system, they could modulate the gut microbiota composition influencing gut microbiota-immune system cross-talk, they could be fermented by the microbiota resulting in metabolites (e.g. short chain fatty acids (SCFAs)) which can act locally on epithelial and immune cells or be absorbed and act systemically, or they could train or prime immune cells involved in surveillance of the luminal contents of the gastrointestinal tract. Although these latter actions are primarily focused on the gastrointestinal tract, because of recirculation of cells from the gut wall to the respiratory tract, effects initiated at the gut level can be enacted at the level of the airways.

## Micronutrients: Support of Normal Immune Function and Relevance for Respiratory Infection

Multiple micronutrients (vitamins and minerals) play several roles in supporting the immune system (**Table 1**), as comprehensively reviewed elsewhere (56, 59–61). People with low intakes or status of these micronutrients show immune impairments and increased susceptibility to infectious disease, especially respiratory and gastrointestinal (56, 60). Amongst the micronutrients, the roles of vitamins A, C and D and the minerals zinc, copper and iron are well explored, but B vitamins, vitamin E, vitamin K, selenium, magnesium and others all have roles.

**Table 1:** Taken from (58). Summary of the effects of various micronutrients on different aspects of immunity.

Micro nutrient	Role in barrier function	Role in cellular aspects of innate immunity	Role in T-cell mediated immunity	Role in B-cell mediated immunity
**Vitamin A**	Promotes differentiation of epithelial tissue; Promotes gut homing of B- and T- cells; Promotes intestinal immunoglobulin A+ cells; Promotes epithelial integrity	Regulates number and function of NK cells; Supports phagocytic and oxidative burst activity of macrophages	Regulates development and differentiation of Th1 and Th2 cells; Promotes conversion of naive T-cells to regulatory T-cells; Regulates IL-2, IFN-g and TNF production	Supports function of B-cells; Required for immunoglobulin A production
**Vitamin B6**	Promotes gut homing of T-cells	Supports NK cell activity	Promotes T-cell differentiation, proliferation and function, especially Th1-cells; Regulates (promotes) IL-2 production	Supports antibody production
**Vitamin B9 (Folate)**	Survival factor for regulator/T-cells in the small intestine	Supports NK cell activity	Promotes proliferation of T-cells and the Th1-cell response	Supports antibody production
**Vitamin B12**	Important co-factor for gut microbiota	Supports NK cell activity	Promotes T-cell differentiation,, proliferation and function., especially cytotoxic T-cells; Controls ratio of T-helper to cytotoxic T-cells	Required for antibody production
**Vitamin C**	Promotes collagen synthesis; Promotes kerathocyte differentiation; Protects against oxidative damage; Promotes wound healing; Promotes complement	Supports function of neutrophils, monocytes and macrophages including phagocytosis; Supports NK cell activity	Promotes production, differentiation and proliferation of T-cells especially cytotoxic T-cells; Regulates IFN-g production	Promotes antibody production
**Vitamin D**	Promotes production of antimicrobial proteins (cathelicidin, b-defensin); Promotes gut tight junctions (*via* E-cadherin, connexion 43); Promotes homing of T cells to the skin	Promotes differentiation of monocytes to macrophages; Promotes macrophage phagocytosis and oxidative burst	Promotes antigen processing but can inhibit antigen presentation; Can inhibit T-cell proliferation, Th1-cell function and cytotoxic T-cell function; Promotes the development of regulatory T-cells; Inhibits differentiation and maturation of dendritic cells; Regulates IFN-g production	Can decrease antibody production
**Vitamin E**	Protects against oxidative damage	Supports NK cell activity	Promotes interaction between dendritic cells and T-cells; Promotes T-cell proliferation and function, especially Th1-cells; Regulates (promotes) IL-2 production	Supports antibody production
**Zinc**	Maintains integrity of the skin and mucosal membranes; Promotes complement activity	Supports monocyte and macrophage phagocytosis; Supports NK cell activity	Promotes Th1-cell response; Promotes proliferation of cytotoxic T-cells; Promotes development of regulatory T-cells; Regulates (promotes) IL-2 and IFN-g production; Reduces development of Th9 and Thl7 cells	Supports antibody production particularly immunoglobulin G
**Copper**		Promotes neutrophil, monocyte and macrophage phagocytosis; Supports NK cell activity	Regulates differentiation and proliferation of T-cells; Regulates (promotes) IL-2 production	
**Iron**	Essential for growth and differentiation of epithelial tissue	Promotes bacterial killing by neutrophils; Regulates balance of M1 and M2 macrophages; Supports NK cell activity	Regulates differentiation and proliferation of T-cells; Regulates IFN-g production	
**Selenium**		Supports NK cell activity	Regulates differentiation and proliferation of T-cells; Regulates (promotes) IFN-g production	Supports antibody production

IFN, interferon; IL, interleukin; NK, natural killer; Th, T-helper; TNF, tumor necrosis factor.

**Figure 2:**
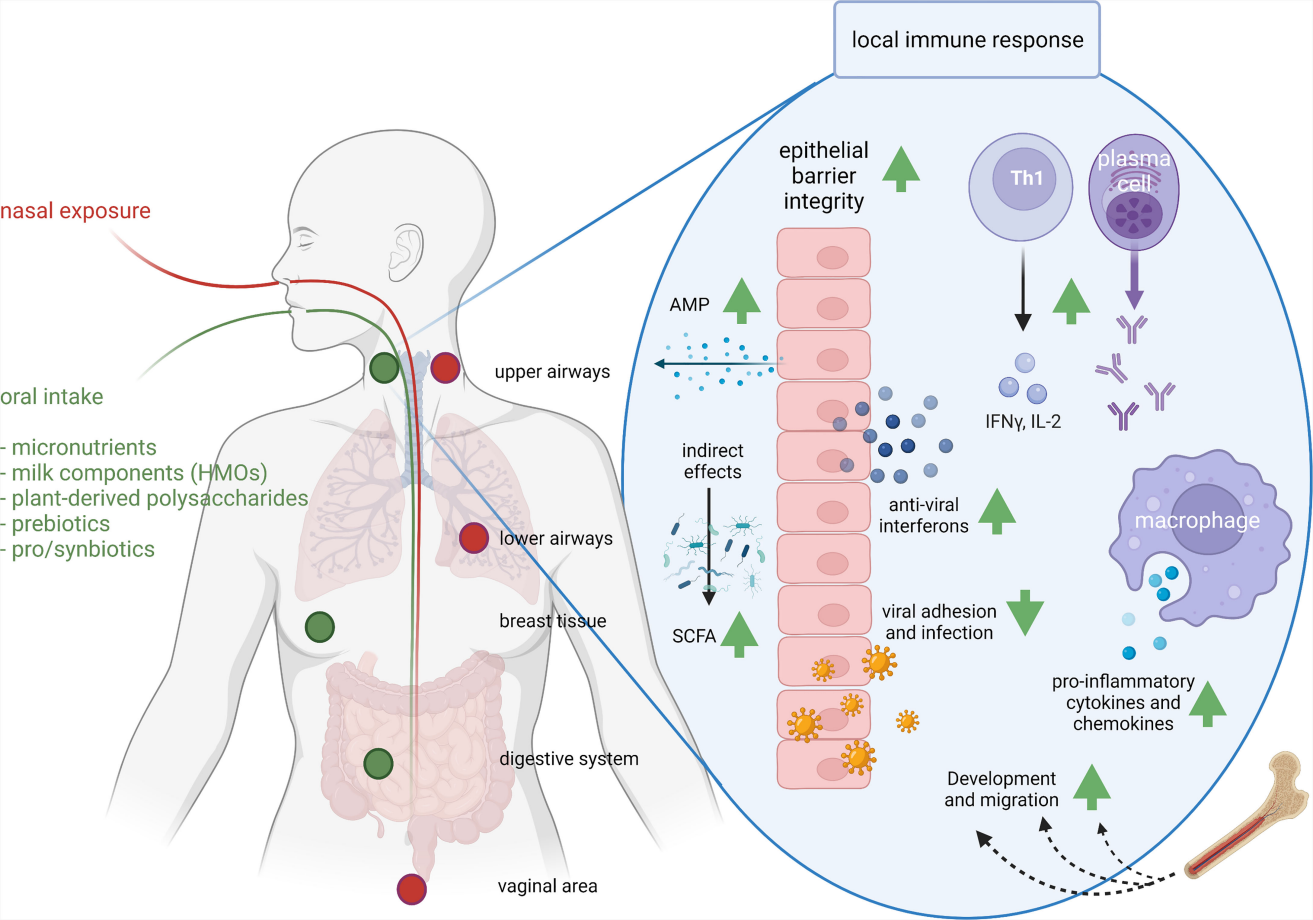
Exposure routes and potential concerted immunomodulatory effects of food components. The route of administration determines the sites of immunomodulation. As a result of nasal exposure (the route that respiratory pathogens follow) pathogens or intranasal vaccines lead to IgA production in upper and lower airways and the vaginal area, and oral exposure leads to IgA production in the upper airways, breast and digestive system. Clinical studies have demonstrated support of respiratory immunity by many food components, whereas preclinical, *ex vivo* and *in vitro* studies identified possible mechanisms. For instance, milk-derived HMOs, pre- and probiotics modify the microbiome and SCFA production; vitamins A, C and D improve epithelial barrier integrity; vitamin D induces anti-microbial protein (AMP) secretion and acetate anti-viral interferon secretion in epithelial cells; viral adhesion and infection is reduced by zinc, bIgG, lactoferrin or HMOs; iron supports immune cell development and migration; bIgG and NDPs induce innate immune training and as a results increased cytokine and chemokine production; zinc and vitamin C support Th1 activity and IFN-γ and IL-2 release; and selenium and vitamin C support antibody production. AMP, anti-microbial peptide; HMO, human milk oligosaccharides; IFN-γ, interferon gamma; IL-2, interleukin-2; SCFA, short chain fatty acid. This figure was created with BioRender.com.

Vitamin A and its metabolites (e.g. retinoic acid) are important for normal differentiation of epithelial tissue and for immune cell maturation and function (62–69). Vitamin A is key to an effective barrier function and it regulates many aspects of innate immunity including neutrophil maturation and function and NK cell activity, thus contributing to defense against viruses including respiratory viruses (**Figure 2**). With regard to acquired immunity, vitamin A controls DC and CD4+ T lymphocyte maturation and promotes Th1 type responses that are involved in antibacterial and anti-viral defenses. The vitamin A metabolite retinoic acid promotes homing of T lymphocytes to the gut-associated lymphoid tissue by upregulating surface homing molecules. In this context it is interesting to note that some gut-associated immune cells are able to synthesise retinoic acid. Retinoic acid is required for CD8+ T lymphocyte survival and proliferation and the normal functioning of B lymphocytes including antibody generation, including IgA. Thus vitamin A deficiency is associated with increased susceptibility to many infections, especially in children, including respiratory infections, diarrhea and severe measles (70, 71). Vitamin A supplementation can improve symptoms in patients with acute pneumonia (69). A meta-analysis of 15 randomized controlled trials in children with pneumonia identified that vitamin A decreased pneumonia morbidity, increased the clinical response rate, shortened clearance time of signs and shortened length of hospital stay, although it did not affect mortality (72).

Vitamin C is required for collagen biosynthesis and is vital for maintaining epithelial integrity (73, 74) (**Figure 2**). It also has roles in several aspects of immunity, including supporting leucocyte migration to sites of infection, phagocytosis and bacterial killing, scavenging reactive oxygen species (ROS), NK cell activity, T lymphocyte function and IFN-γ production (especially by CD8+ cytotoxic T lymphocytes) and antibody production (73, 74) (**Figure 2**). People that are deficient in vitamin C are susceptible to severe viral respiratory infections such as pneumonia and acute respiratory disease syndrome (ARDS) (73, 74). Trials of vitamin C supplementation report reduced risk of pneumonia, with some trials reporting decreased severity and mortality (75). A meta-analysis of trials of vitamin C and common cold infections found that it decreased the incidence in those individuals under physical stress and decreased the duration and severity (76).

Vitamin D receptors have been identified in most immune cells and, besides the occurrence of genetic polymorphisms in the vitamin D receptor gene, vitamin D has pleiotropic actions within the immune system supporting the activity of several cell types (77, 78). Furthermore, some immune cells (DCs, macrophages) can produce the active form of vitamin D suggesting it is important to immunity. Vitamin D enhances epithelial integrity and induces synthesis of antimicrobial peptides (e.g., cathelicidin) in epithelial cells and macrophages (79, 80) (**Figure 2**). Vitamin D promotes homing of immune cells to the respiratory tract (81–84). On a functional level vitamin D promotes differentiation of monocytes to macrophages and increases phagocytosis, superoxide production and bacterial killing by innate immune cells. It also promotes antigen processing by DCs although antigen presentation may be impaired. Thus, vitamin D has many actions which would support immunity against respiratory infections. Nevertheless, the effects of vitamin D on the cellular components of immunity are rather complex and paradoxical in nature as reviewed recently (85). Berry et al. described an inverse linear relationship between vitamin D levels and respiratory tract infections in a cross-sectional study of 6,789 British adults (86). In agreement with this, data from the US Third National Health and Nutrition Examination Survey which included 18,883 adults showed an independent inverse association between serum 25(OH)-vitamin D and recent upper respiratory tract infection (87). A meta-analysis of such observational studies identified that serum vitamin D is inversely associated with risk and severity of acute respiratory tract infections (88). There have been a large number of randomized controlled trials investigating the effect of vitamin D to reduce incidence or severity of respiratory tract infections and these have been subject to meta-analysis. A meta-analysis of 25 RCTs involving over 11,000 adults and children identified that vitamin D decreased the risk of acute respiratory tract infections, with greater effects being observed in those with low starting status (89).

Zinc supports the activity of many cells of the immune system, helps to control oxidative stress and inflammation and has specific anti-viral actions including inhibiting the replication of RNA viruses (90–92). Zinc-binding metallothioneins seem to play an important role in antiviral defense (93). Zinc is important in early immune cell development in the bone marrow and is important in maintaining T and B lymphocyte numbers. Zinc also supports the release of neutrophil extracellular traps that capture microbes. Zinc promotes CD4+ T lymphocyte numbers and function (e.g., IL-2 and IFN-γ production) and supports the proliferation of CD8+ cytotoxic T lymphocytes, key cells in antiviral defense (**Figure 2**). Zinc can disrupt the replication and infectivity of some respiratory viruses and prevents ARDS and lung damage in COVID-19 (94). Zinc supplementation has been reported to improve some markers of immunity especially in older people or those with low zinc intake (95). Recent systematic reviews and meta-analyses of trials with zinc report shorter duration of common cold in adults, reduced incidence and prevalence of pneumonia in children and reduced mortality when given to adults with severe pneumonia (96–98).

Iron is another micronutrient that is required by the immune system for proper functioning. Iron is important to maintain thymus function and the output of naive T lymphocytes and also to support neutrophil respiratory burst and bacterial killing, NK cell activity, T lymphocyte proliferation and production of T helper 1 cytokines (99–101) (**Figure 2**). These observations would suggest a clear case for iron deficiency increasing susceptibility to infection, which it does (102). However, many pathogens also have a high demand for iron and in an acute infection, host-driven iron removal inhibits the growth of pathogens (103–105). Chronic immune activation due to persistent infection, however, sequesters iron not only from infectious agents but also from erythroid progenitors, thereby causing anemia associated with chronic inflammation (106). Therefore, the relationship between iron availability, including deficiency, and susceptibility to infection remains complex (107–109). Evidence suggests that infections caused by organisms that spend part of their life-cycle intracellularly may actually be enhanced by iron, and in such situations providing iron may be harmful. Perhaps because of this harm from iron, several host immune mechanisms have developed for withholding iron from a pathogen (104, 105, 110).

Selenium has important roles in supporting the immune system in general (111, 112) and in promoting anti-viral immunity in particular (113–115). Selenium supports the activity of many cells of the immune system and helps to control oxidative stress and inflammation. Cellular entry of viruses, including SARS-CoV-2, into lung cells causes cellular oxidative stress resulting in the formation of free radicals (ROS) and subsequent disruption of the cellular membrane and lysis because of the budding process. Selenium could protect against such stress. Extensive research in mice has shown that selenium deficiency adversely affects several components of both innate and acquired immunity, including NK cell, T and B lymphocyte function and antibody production (**Figure 2**), increases susceptibility to viral infection, permits viruses (including coxsackievirus, polio virus and influenza viruses) to mutate, and allows normally weak viruses to become more virulent (116–118). A study in South Korea showed that selenium deficiency in peripheral blood was associated with a higher mortality in COVID-19 patients (119). Selenium supplementation improves some markers of immunity especially in older people or those with low selenium intake; for example a supplementation study conducted in UK adults with marginal selenium status showed that selenium improved *ex vivo* anti-viral immune responses, promoted polio virus clearance and decreased polio virus mutation (120). It seems likely that selenium is important in reducing risk and severity of viral respiratory infections.

Taken together, it is evident that multiple micronutrients play several roles in the immune system. Individuals with a deficiency in either one of these vitamins or minerals show immune impairments and increased susceptibility to infection. These impairments can be reversed by repletion of the deficient micronutrient. Each micronutrient has its own biochemical actions that result in its effect on the immune system; although the immunologic effect (*e.g.* improved Th1 cell function) can be shared by many micronutrients, the mechanism underpinning that effect is, generally speaking, unique to each particular micronutrient. In other words, the immune impairments that result from deficiencies in multiple micronutrients are unlikely to be reversed by repletion of only one of those micronutrients due to limited redundancy in function. In such a scenario a multiple micronutrient mixture is likely to be more effective in supporting (impaired) immune function than any single micronutrient.

## Milk Components, Immune Function, and Respiratory Infection

Milk production has evolved in mammals to support growth and development in early life, as well as to provide protection against infections. Therefore, there are many components in breast milk that have direct anti-infective and immune modulatory actions or are important for supporting the immune system (121–131).

### Breastfeeding

The first 1000 days of a child’s life are important for maturing the immune system, partly under the influence of the development of the gut microbiota. Breast milk contains many bioactive components that support the development and can regulate the immune system, as well as components that can have direct antibacterial and antiviral effects. It is therefore not surprising that breast-fed children have fewer infections than bottle-fed children (125, 132, 133). This has been reported for gastrointestinal infections, but also relates to (viral) respiratory tract infections, pneumonia and mortality (134–136).

### Bovine Milk and Its Components

Even though human breastmilk contains many immune supporting components (125, 130), studies on breastfeeding cannot show exactly which components confer resistance to respiratory infections. Cow’s milk contains similar components, often in comparable concentrations (131), and apparently with comparable activity on the human immune system (61, 121, 123, 131). Epidemiological studies have seen an inverse association between the consumption of raw cow’s milk and asthma and respiratory infections (137–140) and have shown that it is heat-sensitive components of raw cow’s milk, likely proteins or peptides, that play an important role (141, 142). In addition, it is known that, like human breast milk, raw cow’s milk contains components that may have antiviral activity like IgG, lactoferrin and lactadherin (131, 143). These are not included in infant formulas in their active form due to structural alterations as a result of heating during processing. Thus, infants that are not breastfed or switch to formula at an early age are at increased risk of infection, in particular when introduced to new foods or an altered environmental exposure to microbes.

Epidemiological research has shown that the consumption of raw cow’s milk by young children is associated with a lower chance of developing asthma and hay fever (137–139, 141, 142, 144–146) and respiratory infections (140). These studies show that heat-sensitive components in cow’s milk - the proteins and peptides - play an important role in protecting against infections and allergies. *In vitro* research has established that milk proteins like IgG, lactoferrin, IL-10 and others can have effects on the human immune system (123, 124, 126, 127, 147–149). These components may contribute to antiviral immunity, but they are not well researched in this context. However, on a cautionary note, it should be stated that raw milk consumption also brings about risks of gastrointestinal infections and therefore consumption of raw milk is not recommended. Heating is applied to kill potential pathogens but also destroys some of the bioactive components of raw milk. Application of these components to support immune function is thus dependent on milk processing that ensures microbiological safety without disrupting their functionality.

### Bovine IgG

Gamma immunoglobulin (IgG) antibodies from cow’s milk can bind to respiratory pathogens such as RSV and influenza virus (148), and also to many bacterial pathogens and to allergens (128). Moreover, cows can be infected by the bovine RSV and thereby induce an anti-RSV response; the IgG antibodies produced can prevent infection of human cells with human RSV *in vitro* (127), and prevent RSV infection in an animal model (147) (**Figure 2**).

The possible role of IgG in relation to other respiratory viruses is not well known. In addition to RSV, cows encounter respiratory coronaviruses (150–152). Cows therefore have coronavirus-specific antibodies, which may also be able to bind cross-reactively to SARS-CoV-2, as can IgA against SARS-CoV-2 from breast milk of infected (153) as well as vaccinated (154) women.

Bovine IgG, like raw cow’s milk, can induce trained immunity (122, 155), a concept that is discussed earlier (**Figure 2**). Through this mechanism, milk IgG antibodies may also provide protection against other pathogens. It is not yet known whether this also works against respiratory infections, but it is known that bovine IgG-induced trained immunity, as well as lactoferrin supplementation in elderly women, enhances subsequent innate immune responses *via* TLR7, a key receptor in immunity against single stranded RNA viruses (122, 155, 156).

### Lactoferrin

Lactoferrin is an iron-scavenging immunomodulatory protein found in milk, but also in serum and other bodily fluids. It has anti-infective effects, including antibacterial and antiviral effects (110, 157, 158) (**Figure 2**). The antiviral properties of lactoferrin present in human breast milk and also in cow’s milk make it a natural supplement with a potential to be used to help protect against respiratory viruses. Lactoferrin’s benefits as a contributor to innate defense, are well documented as supported by a recent meta-analysis (158). Based on the observation that lactoferrin can inhibit infection of cells with the original SARS virus (159), there has recently been a lot of interest in whether lactoferrin can do the same for SARS-CoV-2, but data on this are not yet available (160, 161). It is also known that lactoferrin can induce IFN-λ as an antiviral cytokine in intestinal epithelial cells (40), and can partially restore the deficient response of pDCs of elderly women to TLR7 stimulation (156).

### Other Milk Proteins

Cow’s milk also contains other proteins that may have an effect on the immune system, such as IL-10 (mostly in colostrum), transforming growth factor (TGF)-ß, osteopontin, MFGM8 (lactadherin) and lactoperoxidase. TGF-ß, IL-10 and osteopontin are anti-inflammatory components and do not play a direct role in viral infection, but can help prevent tissue damage arising from infection. Lactoperoxidase and MFGE8 have antimicrobial and possibly antiviral effects (143, 162, 163). These components have not been studied in relation to respiratory tract infection.

### Human Milk Oligosaccharides (HMOs) and Prebiotic Oligosaccharides

As mentioned above, breastfeeding is known to protect against gastrointestinal and respiratory tract infections in infants (132, 164). The concentrations of human milk oligosaccharides (HMOs) in breastmilk are inversely associated with prevalence of gastrointestinal and respiratory tract infections (165), and HMOs and prebiotic oligosaccharides have been shown to enhance immune responses to vaccination or infection (166–171). HMOs play a role in the maturation of the immune system in early life, partly because of their influence on the microbiota composition and the production of microbial metabolites such as short-chain fatty acids (SCFAs) (129, 172, 173). The composition of the intestinal microbiota has been linked to the prevalence of respiratory infections (174–177). The effect of HMOs may in part be indirect *via* an effect on Bifidobacteria (178).

**Figure 3:**
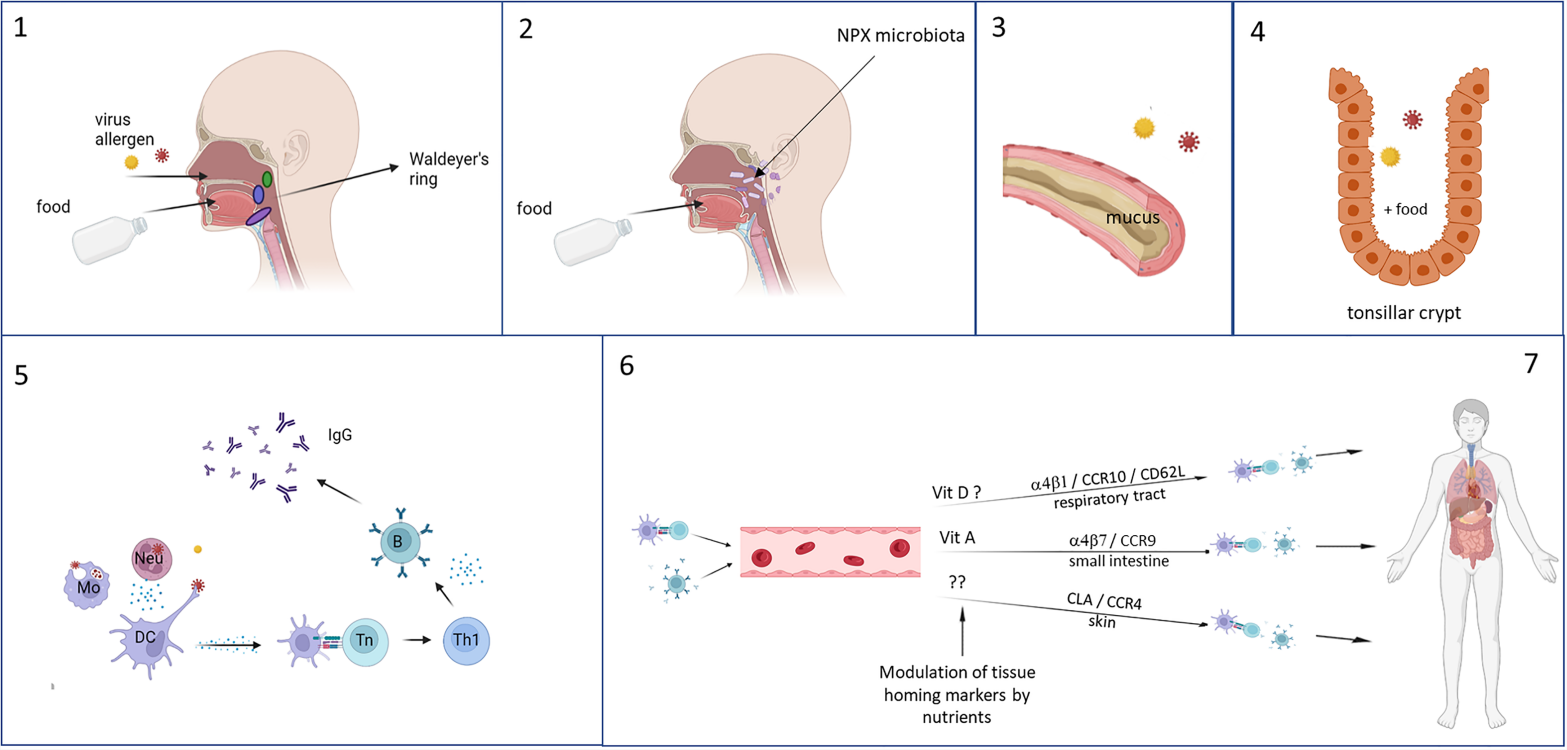
Foods are ingested and enter the gastrointestinal tract *via* the mouth and respiratory pathogens *via* the nasal cavity, both ending up in the tonsils of Waldeyer’s ring (1). Especially in infants, reflux can cause a direct interaction of the foods with the nasopharyngeal microbiota and influences their composition (2). Mixing of the food with saliva and mucus (based on charge and polarity) enhances the residence time in the nasopharynx (3), and as a result the still undigested food and respiratory pathogens can interact with each other and the immune tissues in the tonsil, where stasis occurs in the crypts which further lengthening the duration of interaction (4). Local immune responses can occur, during which can be modulated by the food components (5). Immune cell recirculate and home to different tissues – especially to the upper respiratory tract because of instruction by stromal cells in the tonsils – which is determined by the expression of homing receptors. This can also be influenced by food components, selectively homing, for example by vitamins, resulting in increased memory responses in the local tissues of the upper respiratory tract (6). This figure was created with BioRender.com.

Breastfeeding and early life nutrition may also influence respiratory infections through interaction with the microbiota in the nasopharynx. The composition of the microbiota in the nasopharynx of infants is associated with the occurrence of respiratory tract infections (179, 180) and asthma (181). Furthermore, in breastfed children microbial communities in the nasopharynx are significantly different from those of bottle-fed infants (182), and the early nasopharyngeal microbial composition is linked to reduced risk for respiratory infection (183). These data suggest that breastmilk, and possibly HMOs, may affect the local microbiota composition in the nasopharynx of infants as well as that of the intestinal tract (**Figures 2**, **3**).

Although most of the oligosaccharides in breastmilk are fermented by bifidobacteria and other bacteria in the intestine or are excreted, a small fraction of these HMOs can enter the circulation (184–186), as do the SCFAs that are produced after fermentation - especially acetate and to a lesser extent propionate and butyrate (186, 187). These SCFAs are also induced after the fermentation of prebiotic oligosaccharides like galactooligosaccharrides (GOS), fructooligosaccharrides (FOS) and inulin, as previously reviewed in (188).

SCFAs have been shown to protect against the development of asthma (189–191), suggesting that they might also influence immune responses to respiratory pathogens. Indeed, Trompette et al. showed in a murine influenza model that a high fiber diet and resulting increased intestinal SCFA levels led to decreased neutrophil recruitment to the airways preventing tissue damage in acute infection, as well as enhancing CD8+ T cell responses (192). Recent evidence suggests that SCFAs play a role in the control of respiratory infections through direct action both on microbiota and on host immune signaling (193). Influenza infection itself can also alter the microbiota composition (194), which leads to decreased SCFA production enabling bacterial superinfection by a reduction in the bactericidal activity of alveolar macrophages (195).

In addition to these indirect effects of HMOs *via* the microbiota, sialyllactose (SL) that is also present in bovine milk, has long been known to inhibit the binding of human influenza haemagglutinins to target cells (43, 44, 196, 197) (**Figure 2**). Likewise, HMOs that prevent bacterial adhesion to the respiratory epithelium may play a role in preventing bacterial pneumonia, as was shown for the HMO Lacto-N-Neotetraose (LNnT) in an animal model of pneumococcal pneumonia (198).

At the level of the respiratory epithelium, the HMO, 2’fucosyllactose (2’FL) could decrease the epithelial viral load and cytokine production after RSV exposure, and LNnT and 6’SL reduced viral load and cytokine production of epithelial cells after exposure to influenza virus (45), suggesting that these HMOs prevent infection of epithelial cells by these viruses directly by preventing adhesion. Interestingly HMOs can also enter the amniotic fluid (199), and may, after ‘inhalation’ of the fluid by the fetus, protect against respiratory infections like RSV and influenza in the first days or weeks after birth, as was also proposed for amniotic fluid antibodies by Jacobino et al. (200).

To date several studies have shown a reduction in respiratory infections by inclusion of prebiotics in infant formulas (201–205), and GOS supplementation was reported to reduce the duration of the common cold in healthy students (206). As HMOs have only recently been introduced into infant formulas, so far only a single study has addressed their effect on respiratory infections. In an infant study with formula supplemented with 2’FL and LNnT, parents reported reduced bronchitis, lower respiratory tract infection and less antibiotic use (207). In a follow-up study, infants without bronchitis or lower respiratory tract infections had increased levels of acetate and B. longum subsp. infantis in their stools, which suggests a causal relationship between HMOs, SCFAs and protection against bronchitis (178).

## Plant-Derived Non-Digestible Polysaccharides, Microbiota, Immune Function and Respiratory Infection

Non-digestible polysaccharides (NDPs) constitute a large group of molecules that are found in many different foods and have been demonstrated to broadly and effectively support health and immune activity (208). NDPs include ß-glucans, pectins, resistant starch, arabinoxylans and many other types of NDPs extracted from plants (e.g. ginseng, carrot, oat), fungi (e.g. *Saccharomyces cerevisiae, Lentinula edodes*) or bacteria (e.g., *Alcaligenes faecalis*). Many *in vitro* and preclinical studies have demonstrated the immunomodulatory potency of NDPs [expertly reviewed by Ferreira and colleagues (209) and Jin and colleagues (210)], and clinical studies confirmed some of these findings. Double-blind placebo controlled clinical studies have demonstrated that oral intake of some NDPs (e.g. β-glucans) reduces incidence and duration of upper respiratory tract infections (URTIs) in elderly subjects or athletes (211–214).

Athletes typically demonstrate enhanced frequencies of symptoms of URTIs following intensive exercise (215). Several studies were performed to investigate whether NDP administration could lower incidence of URTIs in athletes. In a first study these subjects were provided with an insoluble ß-glucan derived from the mushroom *Pleurotus ostreatus* (211). A daily intake of 200 mg ß-glucan mixed with 200 mg vitamin C was compared to the placebo intake of only 200 mg vitamin C. During three months of supplementation, subjects in the test group reported a total of 65 episodes of URTI, which was significantly less than the 117 episodes reported by subjects in the placebo group. These results might have been related to the reduced drop in phagocytic potential of PBMCs and increased frequency of NK cells in the PBMCs. In a similar setting, healthy marathon runners were provided a dairy drink as placebo or a dairy drink containing 250 mg per serving per day of insoluble or soluble ß-glucan from yeast for 91 days (213). URTI symptoms were scored according to a validated questionnaire and confirmed by a physician. The total severity of URTI symptoms over the test period was significantly lower for the subjects provided with the insoluble ß-glucan. A final study involving athletes also investigated the administration of insoluble ß-glucan from yeast (216). The ß-glucan was provided in capsules, as was the rice flour placebo, at a dose of 250 or 500 mg per day for four weeks; a significantly reduced number of subjects reported URTI symptoms after two and four weeks of β-glucan when compared to the control group.

In other study population (with older people) similar effects were observed, demonstrating that the beneficial effects are not related to this specific metabolism or physical state of athletes. Older people with an approximate age of 59 years (212) or women selected according to experiencing moderate levels of stress (217) where provided with 250 mg per day of an insoluble yeast ß-glucan for 90 days or rice flour as placebo in capsules. In the older people, the intake of ß-glucan resulted in a trend towards fewer illness episodes and fewer days of illness and in women with above average stress levels β-glucan resulted in a significant reduction in days of symptoms and fewer subjects with URTI symptoms during the test period. Similarly, ß-glucan supplementation in healthy adults reduced severity and duration of URTI symptoms (218, 219).

With ginseng extracts enriched for NDPs, it has been shown in a number of clinical trials that addition to food resulted in support of immune function (220–222) and reduced incidence and severity of viral respiratory infections (223, 224). Research has shown that so-called RG-I fibers (Rhamnogalacturonan-I, referring to specific domains of pectins) are responsible for this effect of ginseng. It has now become clear that RG-I fibers from various plant sources have a modulating effect on immune function and on the microbiota (225, 226). A recent randomized controlled trial explored a low or a higher dose of cRG-I from carrot as a dietary supplement in heathy volunteers (227). After 8 weeks, the volunteers were infected with a low dose of rhinovirus in their nasal cavity and then the immune response and the development of cold symptoms were monitored. Such studies in which the effect of a nutritional intervention on the course after a (viral) challenge is measured in humans are seen as particularly relevant by experts and regulators (228, 229). Consumption of cRG-I was found to greatly accelerate the anti-viral IFN response in infected nasal epithelium and the local innate immune response, resulting in a significantly milder clinical course with less severe and shorter cold symptoms (227).

Fluorescently labelled NDP particles, being either ß-glucan capsules or particulate ß-glucan, were demonstrated to reach the ileum, colon, liver and lung and macrophages in spleen, lymph nodes and bone marrow upon oral administration in mice (230–233). In a different study, radiolabeled NDPs were shown to reach blood, intestine, and spleen and accumulated in tumors in mice (234). These studies confirm that NDPs interact with immune cells of the intestine and that they do not only move to the colon for fermentation. Moreover, these studies show that NDPs can become available in distant peripheral tissues where they can interact with local APCs and exert their immunomodulatory effect. There is clear evidence that molecular patterns on NDPs are recognized by PRRs of the immune system like Dectin-1 while actively “sensing” the contents of the gut (235, 236). This recognition and the subsequent innate immune training or direct immune activation are manners through which NPDs could modulate peripheral macrophage activity (**Figure 2**). Human trials to investigate induction of innate immune training so far were limited to i.v. introduction of NDPs rather than oral intake. To date only a single clinical trial has been performed to investigate orally mediated innate immune training by NDPs. Unfortunately, in this study ß-glucan was barely detectable in blood and blood-derived immune cells did not demonstrate signs of innate immune training (237). Over the past decade many NDPs, including arabinoxylans, ß-glucans and pectins, have been tested for their potency to activate innate immune cells. NDP exposure to peripheral blood monocyte-derived macrophages, either anti- or proinflammatory, or pro-tumorogenic (i.e. TAMs) induced a distinct transcriptional profile (238) and a secretome rich in chemokines and increased co-stimulatory receptor expression (239, 240). Of note, studies have demonstrated that NDP characteristics of solubility, size, monosaccharide composition and side chains (226, 239, 241) were crucial to induce immunomodulation *in vitro* or *ex vivo*.

Taken together, NDPs have been demonstrated to modulate immune responsiveness beyond the GI tract and to support APCs in expressing co-stimulatory receptors and releasing pro-inflammatory cytokines through training and in addition improve recruitment of additional immune cells.

## Pro and Synbiotics

The gut and airway microbiota are linked to respiratory tract health and immunity (‘gut-lung axis’) and modulation by pharmacological agents and pro- and synbiotics has in some studies been shown to manage RTIs (242). One implication of the dynamic bidirectional relationship between gut microbes and the immune system is that changes in the composition and metabolic activity of the gut microbiota can alter immune function, potentially altering host susceptibility to infection including within the pulmonary system.

Probiotics are defined as live microorganisms that, when administered in adequate amounts, confer a health benefit on the host (243). The most commonly used probiotics are Lactobacillus and Bifidobacterium species, followed by the genera Streptococcus, Enterococcus, Propionibacterium, Bacillus, and Escherichia coli (244, 245). In addition, some yeast species are used as probiotics, for example, *Saccharomyces boulardii* and *Saccharomyces cerevisiae* to treat gastrointestinal disorders (246, 247). Generally, probiotics do not extensively divide and therefore do not permanently colonize the gut. In the gut of children, probiotics can persist for up to 4 weeks (248, 249) as they have a relatively stable ecology, while in the gut of adults probiotics persist for only around a week after consumption (250).

Besides their well-described effects in the gastrointestinal tract and in allergic diseases, probiotics also display clinical effectiveness in respiratory tract infections and support lung immunity and inflammation in both children and adults (251). Clinical studies have shown that certain probiotic strains help to prevent bacterial and viral infections, including gastroenteritis, sepsis, and RTIs (252) although others did not find such effects (253). This dynamic bidirectional relationship between gut microbiota and the immune system is sensitive to changes in the composition and metabolic activity of the gut microbiota. This also influences the pulmonary system and alters general host immune competence and thereby susceptibility to infection.

The potential for the use of probiotics has been shown in different studies including a double-blind placebo-controlled trial, in which milk was supplemented with the probiotic *Lactobacillus reuteri*, but not *Lactobacillus casei*, which showed that acute infectious diarrhoea could be reduced in Indonesian children with a low nutritional status (254). Probiotics such as Lactobacillus and Bifidobacterium have shown direct potential in respiratory infections as illustrated in a meta-analysis in which they were able to increase the efficacy of influenza vaccines in adults (255). These probiotics enhanced the rate of seroconversion in older individuals and thereby have potential to be used as preventive treatment before seasonal influenza vaccination. In a systematic review it was shown that the use of selective probiotics and synbiotics in optimized formulations can provide prophylactic and complementary treatment benefits in patients with RTIs, including COVID-19 and influenza infections as they reduce the severity of the infection symptoms and the duration of disease (256).

Probiotics, including *Lactobacillus plantarum* and *Lactobacillus reuteri*, are mainly used to prevent infections and for recovery from gastrointestinal infections and are not yet widely used to prevent or treat URTIs, although some studies have shown them to alleviate symptoms of URTIs by strengthening the respiratory mucosa (257, 258). In recent meta-analyses of double-blind, randomized, and placebo-controlled trials it was shown that probiotics significantly reduced rates of acute URTI and the associated antibiotic use, but they did not decrease the duration of each single infection episode (259, 260). Children that were supplemented with probiotics had fewer days with URTIs and therefore had fewer days absent from day care or school compared with children receiving placebo (234, 245).

Probiotics can modulate the plasma levels of cytokines and exert differential modulation of innate and the adaptive arms of the immune response based on host (epi)genetic differences and the particular microbial strain (251, 261). Dietary supplementation with probiotics like *Bifidobacterium longum*, can stimulate trained immunity in alveolar macrophages, putatively by the NOD2 receptor and the action of histone 3 lysine 4 trimethylation (H3K4me3) to induce epigenetic changes at the level of histones. These macrophages respond more efficiently with increased IFN-γ production to respiratory pathogens like influenza as observed in mice that received these beneficial microorganisms. In these mice, NK cell activity was significantly increased, resulting in enhanced levels of IFN-γ, IL-2, IL-12, and IL-18 in both spleen and lungs resulting in decreased virus proliferation and suppression of inflammation (262). At the same time, the probiotic *Bifidobacterium longum* significantly reduced influenza-infection induced production of pro-inflammatory cytokines, including IL-6 and TNF-α.

Probiotics therefore may promote resistance against pathogens (261). Probiotics increase leukocyte, neutrophil, and NK cell counts and activity while increasing the production of IL-10 and decreasing production of pro-inflammatory TNF-α, IL-1ß, and IL-8 (263). It has been shown that probiotics inhibit Th17 differentiation and the production of IL-17F, IL-23 and TNF-α, while promoting the IL-10 secreting Treg subset (246). Thereby, probiotics inhibit inflammatory responses and regulate immune cell homeostasis. In addition, probiotics increase the frequency of IgA-positive cells in Peyer’s patches in the lamina propria and thereby inhibit bacterial adherence to epithelial cells, maintain the epithelial barrier function, and facilitate the neutralization of bacterial toxins (264). Because IgA-positive B-cells were shown to be able to migrate to other mucosal tissues in the body, their potential to provide protection in the respiratory tract should be investigated further.

Likely candidates responsible for the functional impact of the microbiota and probiotics are SCFAs. Through their activity as histone deacetylase (HDAC) inhibitors, SCFAs might also induce innate immune memory. Mainly butyrate and propionate, and to a lesser extend acetate, demonstrate this inhibitory activity. SCFAs have been demonstrated to affect the immune system in the respiratory tract, for example with influenza infection (192), but also RSV infection (265). In turn, this also means that fibers and complex sugars that lead to the growth of healthy bifidobacteria and lactobacilli, as well as the production of SCFAs, can be important for antiviral immunity. Moreover, production of SCFAs also induced enhanced expression of respiratory IFN-β thereby increasing the expression of interferon-stimulated genes (ISGs) in the lung and thereby protected against RSV-induced disease involving activation of the membrane receptor GPR43 (265).

A profound imbalance in gut microbiota can drive the progression of COVID-19 symptoms towards the acute respiratory distress syndrome (ARDS), and this development might be reinforced by the use of antiviral medication (266). Respiratory infectious diseases might thus be affected by gut dysbiosis characterized by a decline in beneficial commensals and enrichment of opportunistic pathogens (242, 267). Administration of a *Lactobacillus plantarum* 06CC2 in mice with influenza-like disease, decreased expression of pro-inflammatory cytokines and increased the mRNA level of IFN-β, IFN-γ, OAS1a, and ISG15 in the lungs underscoring the antiviral activities of this probiotic (268). Bradley et al. showed that the intestinal microbiota influences IFN-α/β receptor expression on the surface of respiratory epithelial cells, which in the case of respiratory virus infection are able to respond more efficiently to type I IFNs stimulation with enhanced ISG levels and suppressed early virus replication (269). Some studies suggested there is transfer of bacterial strains like *Lactobacillus plantarum* from the gut to the lungs upon development of COVID-19 (270). Next to these demonstrated effects, probiotics are also anticipated to dampen the cytokine storm observed in COVID-19 patients, especially in severe stages of the disease, which was associated with increased levels of cytokines and chemokines like IP-10, MCP-1, MIP-1α, TNF-α, IL-1β, IFN-γ, IL-4 and IL-10 (268).

Probiotic supplementation could inhibit the COVID-19 cytokine storm by simultaneously boosting innate immunity and avoiding the exaggeration of adaptive immunity by the suppression of the inflammatory cytokine response. This way, probiotics can prevent the severity and the occurrence of ARDS. Taken together, modulation of gut microbiota by probiotics is suggested as a possible strategy to improve the clinical manifestations of respiratory tract infections and crucially also of COVID-19 (271).

Probiotics and prebiotics, as described above, individually have roles in improving immune health and in combination (synbiotics) might even synergize. The use of synbiotics, often a combination of *Bifidobacterium breve, Lactobacillus casei*, Streptococcus and galacto-oligosaccharides (GOS) or fructo-oligosaccharides (FOS) and inulin, is primarily based on the assumption that the included prebiotics enhance the survival of the probiotics present in the supplement in the gut as well as stimulating indigenous anaerobic bacteria. Gut microbial components and metabolites (postbiotics) including SCFAs, for instance, are involved in gut-lung immune communication (272).

As indicated earlier, the elderly population is particularly affected by a decline in immune function including the antibody response to influenza vaccination although in this population no beneficial effects in reversal of immunosenescence by synbiotics were found (273). In contrast, a synbiotic (Lactocare^©^) consisting of a mixture of *Lactobacillus rhamnosus, Lactobacillus casei, Lactobacillus acidophilus, Bifidobacterium breve, Lactobacillus bulgaricus, Bifidobacterium longum, Streptococcus thermophilus* (each at 10⁹CFU) plus fructooligosacharide FOS was shown to reduce episodes of viral infection in asthmatic children (274).

A recent systematic review and meta-analysis identified 16 studies including 10,443 participants examining the effects of synbiotics on RTI incidence, duration, and/or severity. Results demonstrated that synbiotic interventions reduced the incidence rate of RTIs and the proportion of participants who experienced a RTI by 16% (275). Three separate large randomized controlled trials compared similar synbiotic supplements (containing 3 to 5 strains of *Lactobacillus plantarum, Lactobacillus rhamnosus*, and *Bifidobacterium lactis*, lactoferrin and prebiotics such as either FOS (short-chain fructooligosaccharides) or GOS (galactooligosaccharides)) to placebo, and all three of these trials showed reductions in the incidence, duration, and severity of RTI with the synbiotic (276). It was also proposed that this synbiotic could reduce SARS-Cov2 infection in high-risk medical staff working in COVID-19 hospital wards (277).

Such synbiotic interventions are suggested to be most effective in the prevention phase of a respiratory infection while they are mostly unable to influence the course of the infection once the pathogen has started to replicate in the host. They are therefore more a preventive than a therapeutic option. Nevertheless, a review describing mechanisms whereby probiotic and prebiotic interventions may link the gut microbiota to the outcome of COVID-19 infection provided arguments that synbiotics may help patients after infection with SARS-COV-2 (278) and this was based on clinical evidence indicating that modulation of the gut microbiota can positively influence COVID-19 progression. The consumption of synbiotic products may thus reduce incidence, duration and severity of respiratory tract infections (279). It would be very interesting to see if the consumption of pre- pro- or synbiotics – or other components discussed in this review - can partially reverse the loss of smell, that is associated with a loss of appetite, and hinders recovery after SARS-CoV-2 infection.

## Anatomical and Physiological Aspects of Interaction of Nutrition With the Immune System in the Respiratory Tract

Micronutrients, milk-derived components, plant derived NDPs and pre-, pro- and synbiotics have all been demonstrated to support anti-viral immunity in *in vitro* and *in vivo* animal models as well as in some clinical studies.

Likewise, oral vaccines can lead to immune responses in the airways, suggesting that the oral route of administration is interconnected with the upper airways. Oral immunization with cholera toxin results in specific IgA being produced in the upper airways, the lymphoid tissues in the head/neck area, the digestive system, as well as in breast tissue [(280) and **Figure 2**]. These data indicate that oral intake of cholera toxin can induce immune responses that also target the upper respiratory tract. Several studies have since then confirmed that oral administration of antigens result in IgA production in the upper respiratory tract (281–283).

However, there appears to be a knowledge gap on how nutritional components in the gastrointestinal tract can affect (viral) infections in the respiratory tract. Here, we introduce a hypothetical model of how many of these food components can have an effect on respiratory tract infections, keeping in mind the anatomy of the GI and respiratory tracts, interaction sites of food and food components with the immune system, and the interplay with the microbiota.

Nutritional components that are absorbed in the GI tract can exert their effects on the local immune system, but *via* systemic circulation also at distant immune sites. Mostly non-absorbed nutrients can have local effects, or can have distinct effects through instructing migratory cells of the immune system, or induction of microbial metabolites, that can become available in the circulation and can reach distant tissues. These local effects can occur before the food reaches the stomach and is digested, and can, in addition to immune effects, have anti-adhesive effects on swallowed respiratory pathogens.

The nasopharyngeal mucosa is an environment that is heavily populated with commensal organisms, some of which are associated with increased or decreased risk for respiratory infections (179, 180, 183). As discussed in the oligosaccharide section, breastmilk-derived HMOs can influence the microbiota in the nasopharynx, thus reducing the risk for respiratory infections *via* a local effect (182).

Because of their anatomical location, the immune tissues of Waldeyer’s ring could have a crucial role in developing potent immune responses to respiratory pathogens (**Figure 3**). The immunological tissues in the head/neck area are mainly structured in tonsils, lymph nodes and lymph follicles. The largest area of contact between food and nasal contents and the immune system occurs in the area where nose and mouth come together, the nasopharynx. The pharynx is surrounded by a number of structured lymphoid nodules (tonsils), comprising the lingual tonsil, the nasopharyngeal tonsils (adenoids), the palatine tonsils and tubal tonsils. The tonsils contain large numbers of cells (1 x 10^9^ lymphoid cells) mainly consisting of B-cells and CD4+ T-cells (284). Together these are named Waldeyer’s ring, also referred to as the nasal mucosa associated lymphoid tissue (NALT).

The tonsils and adenoids have multiple antigen-retaining crypts, that greatly increase the surface area of contact between food components and the underlying cells. The reticulated epithelium lining the tonsillar crypts is probably an antigen entry portal, specialized in transporting antigens to the subepithelial tissue within on average about 10 minutes after food consumption (284–286). The adenoids, but to a much lesser extent the palatine tonsils, have epithelia that have well developed tight junctions, and that also contain M-like cells and DCs that contribute to antigen sampling (287, 288). Because of the anatomical structure and location of Waldeyer’s ring, it functions as a gatekeeper for respiratory, swallowed pathogens from nasal secretions. Likewise, it is also exposed to nutritional components that locally can modulate the immune responses to respiratory pathogens sampled in Waldeyer’s ring (**Figure 3**).

As stromal factors determine tissue homing properties of locally activated lymphocytes, it has been suggested that antigens taken up in the tonsils of Waldeyer’s ring will induce the formation of especially IgG-antibody producing memory B cells with homing capacity to lungs, upper airways and peripheral blood (289).

Selective homing of immune cells to the respiratory tract and the intestine is tightly regulated by the expression of chemokine receptors and adhesion molecules (281, 289–292). Of note, expression of homing receptors can be influenced by interactions with food components, which may also influence recirculation and homing properties of immune cells. For example, the vitamin A metabolite retinoic acid induces expression of CCR9 and thus promotes homing to the intestine (293, 294), and vitamin D3 induces CCR10 that is associated with homing to the respiratory tract (81–84). Interestingly, vitamin D3 has also been shown to block the upregulation of retinoic acid-induced gut homing markers (82, 295) and may thus promote homing to the respiratory tract.

A further aspect to consider is the food matrix. The food matrix may affect whether the nutritional components in the food can mix with saliva and the mucus overlaying the nasopharyngeal epithelium and Waldeyer’s ring. This mixing will increase the duration of retention of foods in the oral and nasopharyngeal mucosa. A longer retention time will increase interaction of these food components with the underlying epithelium and cells of the immune system in Waldeyer’s ring.

Two known examples that can promote residence time and mixing with mucus are charge and water in oil emulsions. Mucins have sialic acid and sulfate groups located on terminal side chains on the glycoprotein molecules, as a result of which they are anionic (negatively charged) at neutral pH (296, 297). Positively charged chitosan microparticles strongly enhance systemic as well as local immune responses to diphteria toxin when applied orally, and significantly enhance IgG production after nasal administration (298). Similar results were obtained with ovalbumin bound to cationic (i.e. positively charged) maltodextrin nanoparticles (299). In addition, water in oil emulsions and gels are other options to increase mouth and GI tract presence of compounds of interest (300). Thus, positively charged components – and ideally a matrix (particulate or viscous) that surrounds ingredients by a positive charge - can enhance the mixing behavior with mucus and saliva, and are expected to result in enhanced interaction between the food product and the mucosal immune system of Waldeyer’s ring.

The above rationale suggests that foods or food components may exert their immunomodulatory function locally in Waldeyer’s ring, thus explaining their effects on respiratory infections.

## Concluding Remarks

Nutritional components can support immune function against respiratory pathogens *via* several mechanisms, and at several levels as described in this review. Nutritional approaches to prevent infections can thus be relevant to help prevent infections in vulnerable people like infants and the elderly, and can help to reduce the pressure on healthcare systems, especially in the winter months when respiratory infections occur frequently.

## Author Contributions

All authors contributed to the writing, preparation and finalization of the manuscript.

## Funding

As this is a review paper, no research was funded. The authors work affiliations covered the hours spent on writing the review.

## Conflict of Interest

PC has research funding from Bayer Consumer Care and acts as an advisor/consultant to DSM, BASF AS, Cargill, Smartfish, Fresenius-Kabi, Bayer Consumer Care, GSK Consumer Healthcare, Danone/Nutricia, Nutrileads and Kemin. RN is employed by FrieslandCampina. RA is employed by Nutrileads.

The remaining authors declare that the research was conducted in the absence of any commercial or financial relationships that could be construed as a potential conflict of interest.

## Publisher’s Note

All claims expressed in this article are solely those of the authors and do not necessarily represent those of their affiliated organizations, or those of the publisher, the editors and the reviewers. Any product that may be evaluated in this article, or claim that may be made by its manufacturer, is not guaranteed or endorsed by the publisher.
